# A tandem segmentation-classification approach for the localization of morphological predictors of *C. elegans* lifespan and motility

**DOI:** 10.18632/aging.203916

**Published:** 2022-02-25

**Authors:** Evgeniy Galimov, Artur Yakimovich

**Affiliations:** 1 Artificial Intelligence for Life Sciences CIC, London, United Kingdom; 2 Center for Advanced Systems Understanding (CASUS), Helmholtz-Zentrum Dresden-Rossendorf e.V. (HZDR), Görlitz, Germany; 3 Bladder Infection and Immunity Group (BIIG), Department of Renal Medicine, Division of Medicine, University College London, Royal Free Hospital Campus, London, United Kingdom

**Keywords:** aging, deep learning, *Caenorhabditis elegans*, interpretable machine learning, convolutional neural networks

## Abstract

*C. elegans* is an established model organism for studying genetic and drug effects on aging, many of which are conserved in humans. It is also an important model for basic research, and *C. elegans* pathologies is a new emerging field. Here we develop a proof-of-principal convolutional neural network-based platform to segment *C. elegans* and extract features that might be useful for lifespan prediction. We use a dataset of 734 worms tracked throughout their lifespan and classify worms into long-lived and short-lived. We designed WormNet - a convolutional neural network (CNN) to predict the worm lifespan class based on young adult images (day 1 – day 3 old adults) and showed that WormNet, as well as, InceptionV3 CNN can successfully classify lifespan. Based on U-Net architecture we develop HydraNet CNNs which allow segmenting worms accurately into anterior, mid-body and posterior parts. We combine HydraNet segmentation, WormNet prediction and the class activation map approach to determine the segments most important for lifespan classification. Such a tandem segmentation-classification approach shows the posterior part of the worm might be more important for classifying long-lived worms. Our approach can be useful for the acceleration of anti-aging drug discovery and for studying *C. elegans* pathologies.

## INTRODUCTION

The nematode *Caenorhabditis elegans* (*C. elegans*) is an established model for studying various interventions into the aging process, which allowed to find numerous genes and drugs interfering with aging. 5 out of 7 Tier 1 and 4 out of 6 Tier 2 anti-aging drugs considered for human trials extend lifespan in the *C. elegans* model. There are many aging pathways conserved among species and the worms are expected to be used extensively not only in longevity research but also in the appearing anti-aging industry [1]. Additionally, humanized worms are now used to establish promising models for neurodegeneration [2]. However, unlike genetics of longevity, *C. elegans* phenotypes of aging are not well studied yet. Particularly, we know little about age-related pathologies and their development, as well as, which pathologies determine lifespan and how they cause death [3]. Several pathologies including gut atrophy, uterine tumours and pharyngeal infection were described recently [4–6]. In this light, discovering new *C. elegans* pathologies, particularly determining lifespan, is becoming an important challenge. Studying pathologies in *C. elegans* might help to get a better understanding of the aging process, as well as, the mechanisms and effects of anti-aging drugs.

Recent advances in machine learning (ML) and deep learning (DL) [7] may aid aging studies employing *C. elegans* through uncovering and summarizing previously unseen behavioral and morphological patterns in large experimental datasets. For example, in a recent work several physiological parameters were measured longitudinally and an application of support vector regression allowed to explain different amount of variance in *C. elegans* lifespan by: movement (57%), cross-sectional size (5%), texture (42%), autofluorescence (52%), oocyte laying rate (28%) [8]. Interestingly, it was found that the brood size correlates with lifespan in mated hermaphrodite (r = 0.28) [9]. Furthermore, independent studies confirm that the muscle function is probably the best predicting physiological feature: fast pharyngeal pumping span (r = 0.49), and pharyngeal pumping span (r = 0.83) were found to be highly correlated with the lifespan length [10]. Also, maximum velocity at day 9 [11] and the rate of speed decay (days 3–9) [12] predict 71% and 91% of variability in lifespan accordingly. Cellular and molecular predictors of *C. elegans* lifespan length were also discovered. Expression of hsp-16.2 induced by heat shock in day 1 adults was found to be correlated with lifespan [13]. Free of confounding effects of interventions like heat shock, basal expression of sod-3 at day 9 also correlated with lifespan (r = 0.57), which probably reflects response to pathogenic food [14]. Mir-71 expression from day 4 onwards can be highly predictive and explains 47% of variability in lifespan [15]. Strikingly, a strong inverse correlation (r = –0.93) between nucleolar size (measured on day 1) and longevity indicates deregulated protein synthesis as an important component of aging [16]. Noteworthy, early on a Machine Vision approach was also applied to classify aging phenotypes in *C. elegans*. Particularly, linear discriminant classifier was used to segregate images of pharynxes of different ages for subsequent molecular characterization [17].

Among other methods, one of the most powerful machine learning approaches, particularly for image analyses, is the use of convolutional neural networks (CNN) [18], which are inspired by the visual cortex neural network organization. CNN allowed to achieve impressive results in image recognition, with near human performance on MNIST dataset and outperformed humans on traffic sign recognition by a factor of two [19]. CNN repeatedly showed best performance during “The ImageNet Large Scale Visual Recognition Challenge” in image classification [20, 21]. Introduction of skipped connections to CNN dramatically improved their speed and accuracy, and such residual CNNs are now state-of-the-art for image classification [22, 23]. Encoder-decoder residual networks like U-Net [24], V-Net and Tiramisu also outperform the classical boundary extraction, threshold and region-based methods used in the medical image segmentation field [25]. Despite the impressive results with DL approaches, one of the main drawbacks is that DL networks are black boxes so it is difficult to get the features important for decision making by the network [26]. To circumvent this shortcoming, several saliency techniques have been proposed [27–29]. One such technique is using the global average pooling layer to produce a so-called class activation map (CAM) and localize class-specific image regions in an unsupervised manner [30]. The produced generic localizable deep features can aid researchers in understanding the basis of discrimination used by CNNs for their tasks. However, thus far, no approaches to combine biologically meaningful image segmentation and classification saliency to facilitate phenotype discovery through interpretation have been developed.

Remarkably, CNN were recently used to predict lifespan in worms. In the first paper, a dataset of 913 images of *C. elegans* were used. Each time point (day) has at least 30 worms, and all of them were anaesthetized before imaging. InceptionResNetV2-based architecture achieved a mean absolute error (MAE) of 0.96 day in the regression mode, and an accuracy of 57.6% in classification mode [31]. In another work, the authors used an automatic imaging system capable of tracking the same worm during the whole lifespan, so they had data for 734 worms for which images were taken every 3.5 hours. They used U-Net to segment worms from the background and then performed the worm body coordinate regression to create straightened worm representations. Then they used a modified ResNet34 and managed to regress worm age with minimal MAE of 0.6 days for raw images [32].

Here we used the same dataset as in [8, 32], however instead of predicting age of each worm, we develop a CNN-based platform we called WormNet capable of classifying young adults (day 1–3) into short-lived and long-lived, and also design an approach for extracting features important for such classification. Similarly, we have applied WormNet to classify *C. elegans* movement. To interpret classification results in a by-design fashion, we have accompanied classification CNN with a tandem segmentation CNN. For this, we devised a new U-Net-based architecture (HydraNet) for segmenting worms from the background and also segmenting the worm's body into anterior, mid-body and posterior parts. Interpretation of the classification results was achieved through the union of HydraNet segmentation and class activation maps generated using WormNet. The class activation maps analyses combined with body part segmentation in such tandem fashion allowed us to extract features responsible for lifespan prediction. Finally, using higher resolution segmented version of the *C. elegans* images, we verified our results in a higher expressive capacity residual CNN InceptionV3 accompanied by manual interpretation.

## RESULTS

**Figure 1:**
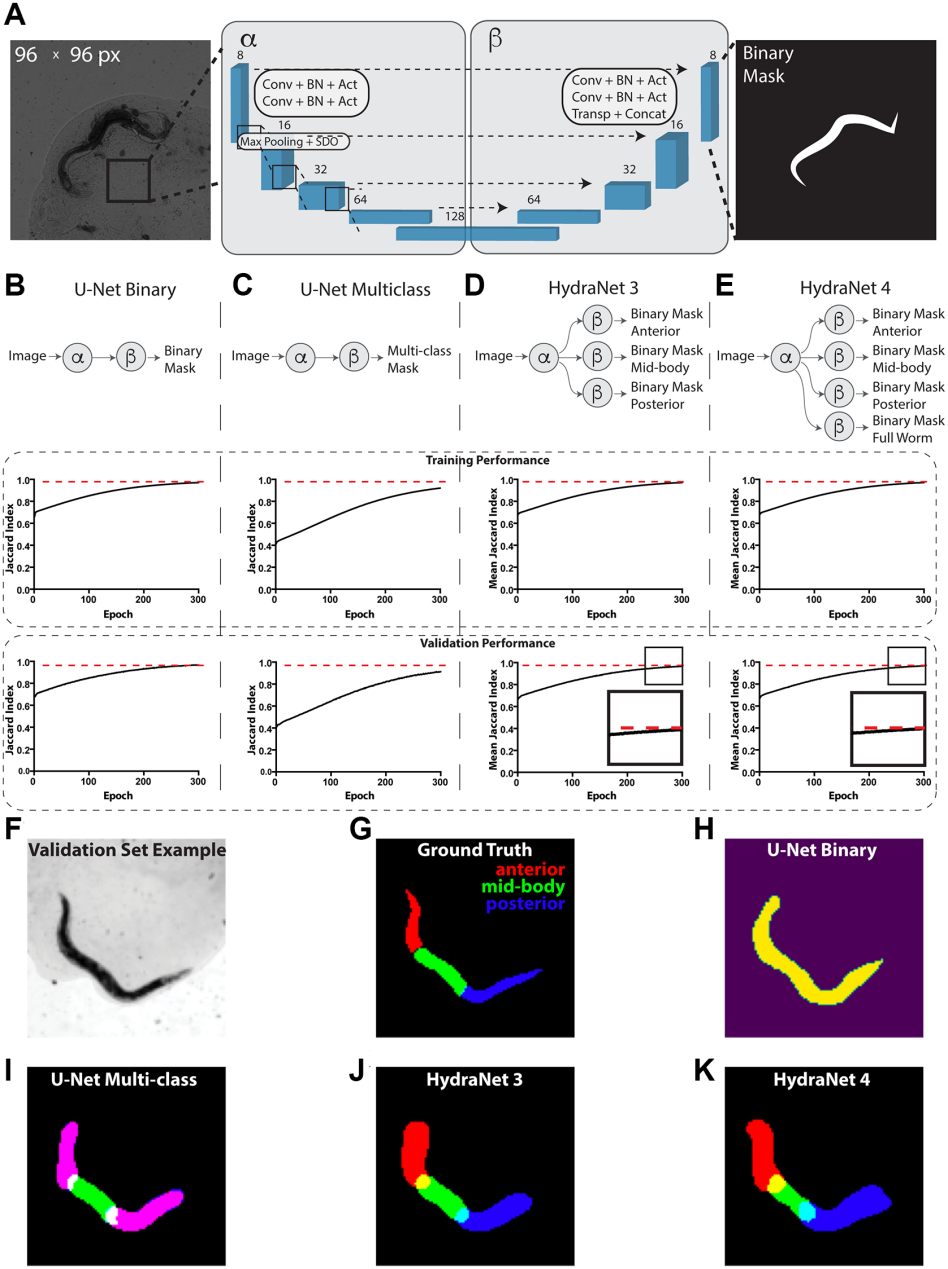
**Devising worm body parts segmentation strategy.** (**A**) Schematic depiction of the U-Net architecture adopted from [24]. Here, the transmission light micrograph of *C. elegans* used as input is depicted on the left-hand side. The reference size of the field-of-view is 580.5 μm by 580.5 μm sized to 96 × 96 pixels. A schematic depiction of a binary mask used as output is depicted on the right-hand side. The displayed numbers correspond to the number of filters in convolutional (Conv), batch normalization (BN) and activation (Act) layers. Max pooling layers were combined with spatial dropout (SDO). Arrows correspond to skip connections from encoder to the mirroring decoder layer where a new layer is a result of concatenation (Concat) of a layer from the encoder part to the transposed convolutional layer (Transp) from the decoder part. For illustration purposes, parts of architecture were grouped into left (α) and right (β) parts. (**B**) Schematic depiction of the binary classification U-Net architecture variant. (**C**) Schematic depiction of the multi-class classification U-Net architecture variant. (**D**) Schematic depiction of the HydraNet 3 architecture variant (**E**) Schematic depiction of the HydraNet 4 architecture variant (**B**–**E**) Here α is the left and β is the right part of the architecture in (**A**). Graphs below show training and validation segmentation performance of the network measured as Jaccard Index for each training epoch. (**F**) Test set input data example. (**G**) Ground truth of *C. elegans* body parts segmentation example. (**H**) Output example of binary classification U-Net on the test data. (**I**) Output example of multi-class U-Net on the test data. Here, red and blue colored masks overlap making anterior and posterior parts appear magenta. (**J**) Output example of HydraNet 3 on the test data. (**K**) Output example of HydraNet 4 on the test data.

The time-lapse data for 734 *C. elegans* captured from day 1 of adulthood till death were used to develop our prototype platform [8, 15]. To develop an approach for automated interpretability of these images we addressed a problem of segmenting the worms from their background, as well as distinguishing worms’ morphological parts (Figure 1). For this, we have manually annotated 130 images of adult worms with masks for anterior, mid-body, posterior parts of the worm and summing up to a total worm mask (Figure 1F–1H). This dataset was then split into the train (90) and test (40) fractions based on the dataset ID of an individual worm to ensure that individual worm features would not leak to the test hold-out. First, to address the total worm segmentation problem we have constructed a relatively shallow architecture akin to U-Net [24] accompanied with a sigmoid head for binary classification. For clarity, the encoding and decoding parts of U-Net are shown on Figure 1A as α and β. The raw images were scaled to 96 × 96 pixels for computational efficiency. We used the Dice loss function and monitored Jaccard index to assess the segmentation quality. On this relatively simple segmentation problem Jaccard index reached 0.97 on both train and test fractions (Figure 1A, 1B, see Materials and Methods for detailed hyperparameters). Next, to extend this approach to segmentation of individual body parts of *C. elegans* we have reformulated the problem as a multi-class segmentation with one-hot encoded masks and similar U-Net-like architecture (Figure 1C, 1I). Unsurprisingly, since a multi-class classification is a harder problem, this led to a worse performance of 0.92 and 0.91 Jaccard index on train and test fraction respectively suggesting a mild overfit.

Remarkably, one aspect multi-class U-Net did not perform well was distinguishing anterior and posterior parts of the worm which led to generating overlapping masks (Figure 1I). To circumvent this limitation, we have designed an alternative architecture using U-Net α and β parts, with multiple β parts dedicated each for its own binary segmentation problem (Figure 1D, 1E), which we called HydraNet. Such approach creates a jointly trained architecture with common input layers and layers dedicated for each of the morphological parts of the worm, allowing to have an end-to-end model, while solving a simpler binary classification problem. HydraNet3 was equipped with 3 β parts dedicated to the anterior, mid-body, and posterior parts of the worm body. HydraNet4, in turn, was equipped with 4 β parts dedicated to the anterior, mid-body, posterior parts as well as the whole worm body. To estimate joint performance of HydraNet we measured Jaccard index for each β part individually and finally evaluated the average Jaccard index. Remarkably, both HydraNet3 and HydraNet4 achieved the average Jaccard index 0.97 on both the train and test fractions demonstrating good generalization (Figure 1D, 1E, 1J, 1K). Noteworthy, HydraNet4 achieved conversion earlier than HydraNet3 (Figure 1D, 1E insets) suggesting a potential positive effect from accompanying the architecture with a more general semantic class.

**Figure 2:**
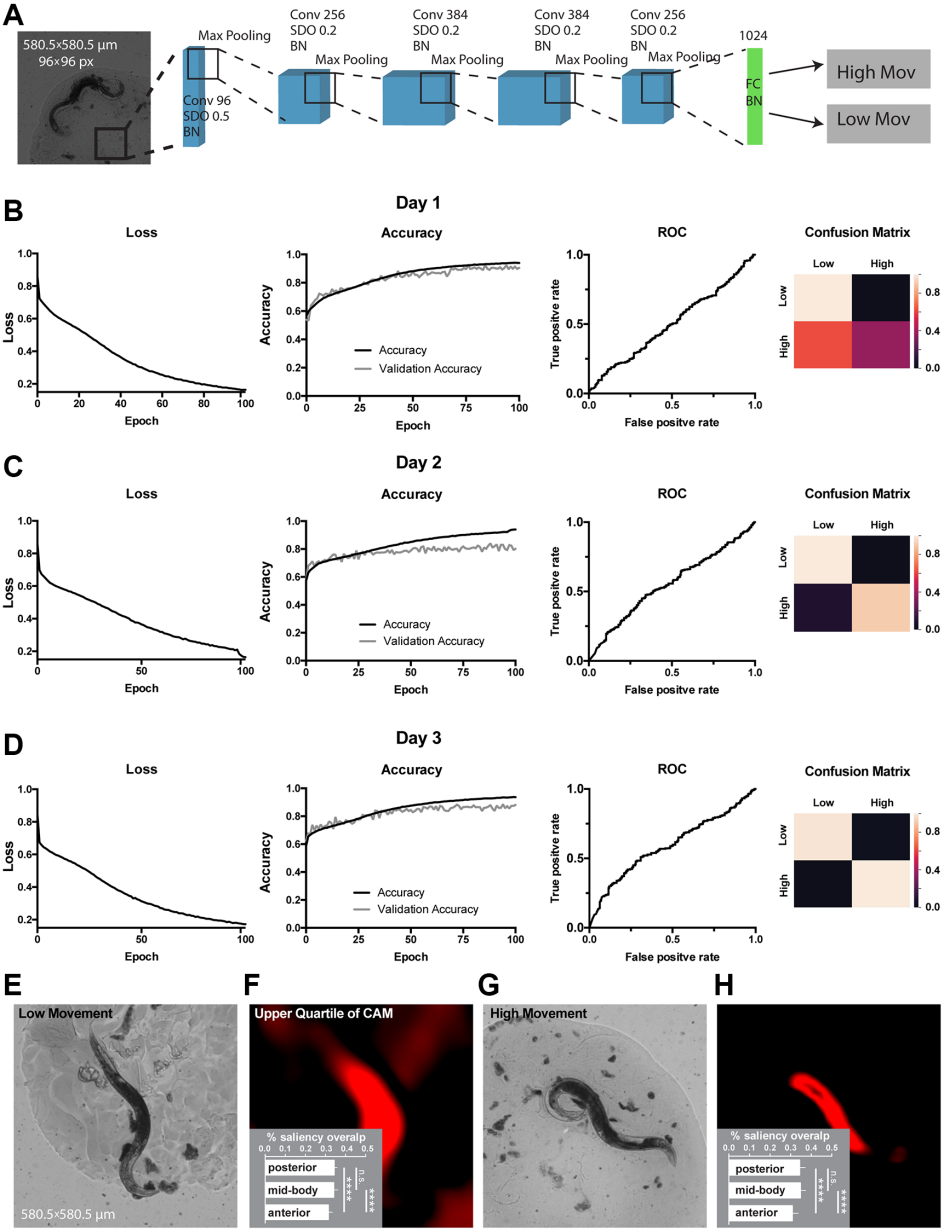
**Classification of movement from end-point *C. elegans* micrographs accompanied by the by-design-interpretation based on segmentation and saliency union.** (**A**) Schematic depiction of the WormNet architecture. Numbers correspond to the number of filters in convolutional (Conv), fully connected (FC), batch normalization (BN) and activation (Act) layers. Max pooling layers were combined with spatial dropout (SDO). (**B**–**D**) End-point day 1, 2 and 3 (respectively) micrographs classification loss (cost function), accuracy, receiver operating characteristic (ROC) curve, and confusion matrix. Training and test (validation) holdouts are depicted as black and light-grey lines respectively. (**E**) Low movement test micrograph example. (**F**) Upper quartile of saliency through class activation map (CAM) from image in E accompanied by the quantified by-design-interpretation using HydraNet 4 and CAM union (% saliency overlap). One-way ANOVA with Tukey’s HSD correction. Mean ± SEM, *p*-value <0.0001. (**G**) High movement test micrograph example. (**H**) Upper quartile of saliency through class activation map (CAM) from image in G accompanied by the quantified by-design-interpretation using HydraNet 4 and CAM union (% saliency overlap). One-way ANOVA with Tukey’s HSD correction. Mean ± SEM, *p*-value <0.0001. Here, the reference size of the field-of-view is 580.5 μm by 580.5 μm.

**Figure 3:**
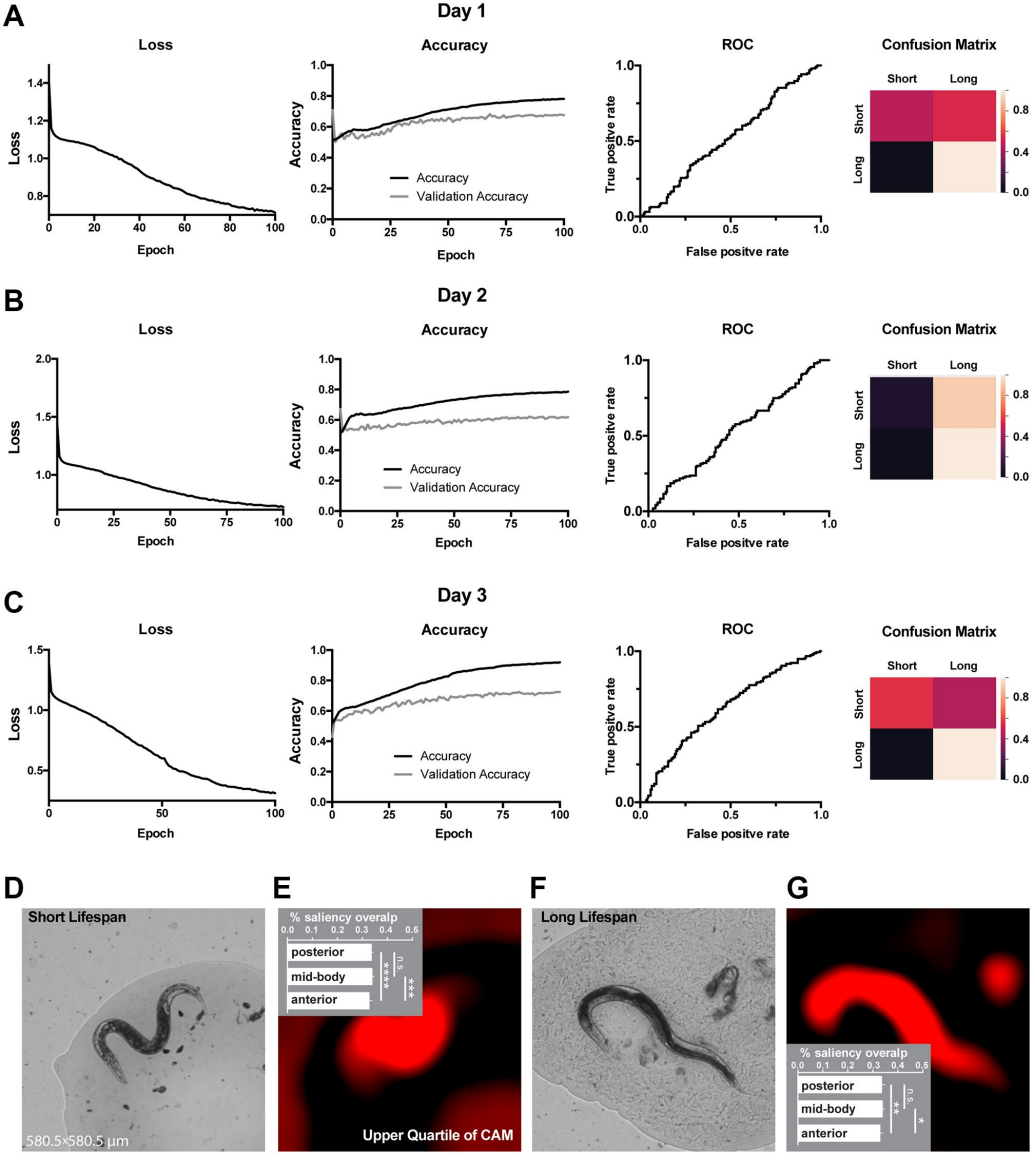
**Classification of lifespan from end-point *C. elegans* micrographs accompanied by by-design-interpretation based on segmentation and saliency union.** (**A**–**C**) End-point day 1, 2 and 3 (respectively) micrographs classification loss (cost function), accuracy, receiver operating characteristic (ROC) curve, and confusion matrix. Training and test (validation) holdouts are depicted as black and light-grey lines respectively. (**D**) Short lifespan test micrograph example. (**E**) Upper quartile of saliency through class activation map (CAM) from image in D accompanied by the quantified by-design-interpretation using HydraNet 4 and CAM union (% saliency overlap). One-way ANOVA with Tukey’s HSD correction. Mean ± SEM, ^***^p-value <0.001, ^****^p-value <0.0001. (**F**) Long lifespan test micrograph example. (**G**) Upper quartile of saliency through class activation map (CAM) from image in F accompanied by the quantified by-design-interpretation using HydraNet 4 and CAM union (% saliency overlap). One-way ANOVA with Tukey’s HSD correction. Mean ± SEM, ^*^p-value <0.05, ^**^p-value <0.01. Here, the reference size of the field-of-view is 580.5 μm by 580.5 μm.

Next, to obtain classifiers for *C. elegans* movement or lifespan, we split all 734 worms into 2 total movement amount classes: low or high movement estimated as motility above or below average distance crawled during the life-time; and 2 lifespan classes: ‘short-lived’ with lifespan 7 days or less, and ‘long-lived’ with lifespan 8 days and more. The task was to predict classes based on day 1, day 2 or day 3 images. As the dataset is relatively small, the use of high expressive capacity architectures could lead to overfitting. Therefore, we designed a relatively shallow CNN we called WormNet. This architecture consisted of 5 convolutional layers, each followed by a max pooling layer. Dropout and batch normalization were implemented for each convolutional layer in the neural network to improve generalization. The last max pooling layer was flattened and attached to a fully connected layer followed by a softmax layer. We used binary cross-entropy as a loss function. All the layers, except the latter one, used a rectified linear unit (ReLU) as an activation function (Figure 2A, see Materials and Methods for detailed hyperparameters). WormNet was used to obtain both movement and lifespan classifiers (Figures 2 and 3). To further alleviate potential overfitting, we performed a 30-fold data augmentation using Keras image generators. Specifically, images are subject to random horizontal and vertical flipping, horizontal and vertical shift within 10% range, as well as random rotations within 90 degrees range of the original. Blanks in the transformed images were filled using the nearest value strategy.

The WormNet showed good performance on total movement classification reaching 88% accuracy (precision 0.86, recall 0.86, area under curve for receiver operating characteristic - AUC ROC - was 0.56) on the test dataset for the day 3 adults fraction. The performance for the day 1 and day 2 images were slightly lower (Figure 2B–2D) with ROC AUC of 0.51 and 0.55 respectively. To ensure our prediction is influenced mostly by the worm morphology rather than its surroundings, we have generated a dataset of synthetic background images where *C. elegans* were removed through segmentation. To alleviate worm silhouette influence on the training, we have filled the remaining zero pixels with random noise (Supplementary Figure 1). Our results suggested that the model performance is predominantly attributed to the *C. elegans* morphology rather than the background of the images. To assess which body part might be responsible for the WormNet decision making, using our tandem segmentation-classification approach we have obtained CAMs for a low movement class worm (Figure 2E, 2F) and a high movement worm (Figure 2G, 2H) from WormNet. Next, each image was segmented using HydraNet4 and the union of WormNet upper quartile CAM with morphological part segmentation from HydraNet4 was obtained. For interpretation purposes we have computed the percentage of CAMs belonging to a respective morphological segment for each respective worm belonging to high or low movement class. Furthermore, we assessed the significance of this by-design interpretation using one-way ANOVA with Tukey’s honest significant difference (HSD) correction (Figure 2F – low movement worms, Figure 2H – high movement worms). The comparison suggested that the anterior part was covered significantly less (31%) than mid-body (34%) and posterior parts (34%) for both low and high movement worms. There was no significant difference between the mid-body and the posterior part of the body.

Next, we used WormNet to classify long and short-lived worms. Similarly to movement classification, the WormNet performed better on day 3 adults sample reaching accuracy of 72% (precision 0.73, recall 0.71, AUC ROC 0.61) on the test dataset, as compared to AUC ROC of 0.53 and 0.52 for day 2 and 1 respectively. The confusion matrix analysis suggested that the CNN underperformed in short-lived worms classifying (Figure 3A–3C). Next, we have interpreted the classifier using the tandem of HydraNet4 and WormNet accompanied by a one-way ANOVA statistical test. In the case of lifespan classification, by-design interpretation suggested that at 32% the anterior part was significantly less pronounced in CAMs compared to the mid-body and the posterior part (Figure 3D, 3E – short lifespan, Figure 3F, 3G – long lifespan). This difference was less significant for long lifespan than for short lifespan. There was no significant difference between the mid-body and the posterior part.

**Figure 4:**
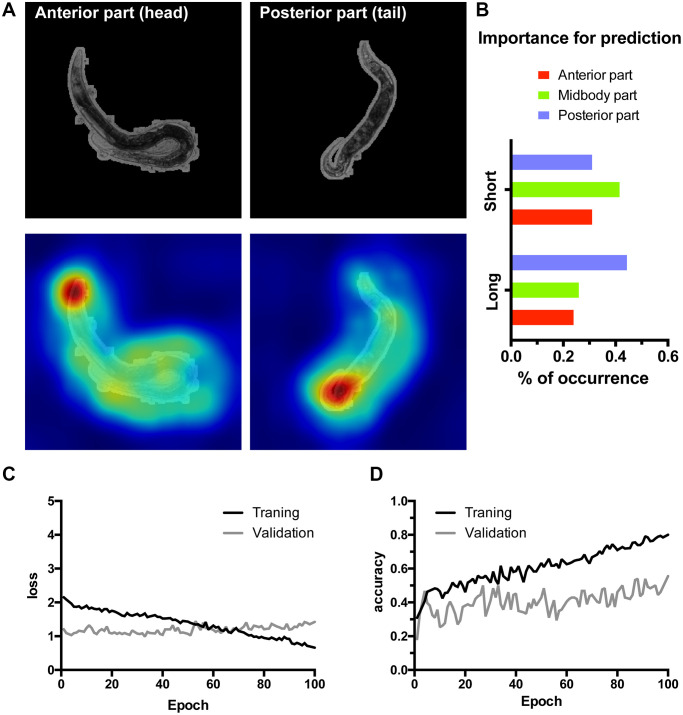
**Class activation maps (CAMs) of the InceptionV3 network overfitted on high resolution images allow localizing lifespan-related regions.** (**A**) Examples of the class activation maps pointing to anterior (left) or posterior (right) parts of a worm. Here 516 by 516 μm area was represented by the 800 × 800 px input image for higher resolution input. (**B**) *C. elegans* body parts importance for class prediction measured as the percent of occurrence of body parts highlighted on the CAMs. (**C**–**D**) Loss and accuracy training statistics for InceptionV3 network.

To verify these findings in an independent manner we have trained another lifespan classifier using the residual InceptionV3 architecture [33] accompanied by a manual interpretation (Figure 4). Furthermore, in this case to ensure high resolution of the CAMs instead of scaling to 96 × 96 pixels, the full resolution 900 × 900 images cropped to 800 × 800 pixels (516 × 516 μm) were used. As a much higher expressive capacity CNN, InceptionV3 was prone to overfitting on our relatively small dataset (Figure 4C, 4D). To circumvent this, we have implemented early stopping during training. Additionally, we segmented the worms from their background ensuring InceptionV3 is presented only with the relevant part of the image. InceptionV3 performed similarly to WormNet with the accuracy reaching 70% on the test dataset for lifespan classification (Figure 4A). Consistently with the tandem HydraNet4-WormNet approach to interpretation, in the case of the manual interpretation, the anterior part of the worm was highlighted by the InceptionV3 CAM less frequently. Importantly, however, due to the higher resolution of the input images, the CAMs now localized the body parts much better, allowing to assign a body part as a possible discriminator in each case (Figure 4B). Interestingly, the distribution of the body parts highlighted by CAM’s analysis demonstrates that the posterior part is more important for long-living worms’ classification, suggesting that the features predicting longevity could be located in the posterior part of the worm body.

## DISCUSSION

Despite *C. elegans* being a classical model in aging research with more than 4000 papers published up to date, and the progress in robotics, the process of measuring *C. elegans* lifespan is still manual and laborious. However, new approaches are emerging like *lifespan machine* utilizing flatbed scanners to simultaneously assess the viability of a large population of worms on plates [34]. Another approach is Worm corals – an automated vermiculture method allowing to track worms throughout their lifespan with much better detailed measurements [8]. The detailed physiological data produced on Worm corals showed that movement, autofluorescence and textural degradation are the best predictors of lifespan. However, it remains unclear what exact morphological features reflect pathologies and determine the lifespan length. It was also found that physiological measurements before day 3 or 4 of adulthood as well as single GFP labelled biomarkers are not able to distinguish short and long-lived worms [8, 15]. Nucleolar-based predictions made on day 1 adults are performed using 100× magnification on fixed worms, which is not achievable for any automated screening platform.

Here we worked with the dataset generated in Pincus lab [8, 15], and showed that the application of newly designed WormNet was able to successfully discriminate between short and long-lived worms even for images taken at day 1 or day 2; importantly, for day 3 the CNN demonstrated the best performance (Figure 2A–2C). WormNet was even better at classifying worms with high and low total movement, achieving 88% accuracy for day 1 adults (Figure 3). We expect that generating more data and developing the CNN architecture could further improve the performance of WormNet by decreasing overfitting and bias.

CNN were used before for regressing and classifying *C. elegans* lifespan [31]. The authors manually classified curvy and straight postures of nematodes which allowed them to improve their accuracy, which still remained relatively low due to small sample size (913 images). Pincus' dataset was also used to assess CNN ability to predict lifespan [32]. As mentioned earlier, the authors segmented the worms and created straightened worm representations, which were used for CNN training [32]. Increased number of samples improved the regression-based prediction of worm age. Interestingly, the authors found that worm silhouette alone has limited information for age estimation, whereas the information from background can significantly improve the accuracy, though the predictive value of background is an artefact of experimental conditions. Therefore, it might be possible that the predictive accuracy of WormNet in our simulations can be partly explained by the background information. However, as our experiments suggest (Supplementary Figure 1), WormNet performance mostly depend on the *C. elegans* morphology rather than the background of the images. Importantly, pretraining on the body-coordinate representations in [32] improved accuracy on raw images which suggests that worm organs and texture are useful for age prediction.

In addition to lifespan or movement classification based on young adults’ images, we also aimed to find features important for the prediction. As a prototype task we decided to determine which body part – anterior, mid-body or posterior part contains features influencing lifespan length the most. We designed HydraNet 3 and 4, new architectures based on U-Net and showed that they can successfully segment worm body parts achieving perfect Jaccard index values. Importantly, to develop a by-design interpretation approach we employed a tandem of biologically meaningful classification (lifespan and movement) yielding saliency through class activation maps [30, 35] and morphological segmentation (anterior, mid-body and posterior regions) to find which body part is useful for the classifications. Furthermore, although less resolved, findings obtained from the tandem approach were consistent with an independently trained classifier. This binary classifier was based on the InveptionV3 CNN. It was trained on 800 × 800 pixels full optical resolution images with worms segmented from their background and achieved results comparable to WormNet, though the model is less generalizable due to more overfitting (Figure 4). However, in the case of InceptionV3, distinct body parts could be localized on the CAMs, and the analyses suggest that features located in the posterior part of the worm might be more important for classifying long-lived worms.

This approach provides an avenue to the discovery of new important age biomarkers in *C. elegans* in an automated setting, given a significant increase in image resolution and usage of body-coordinate representation. Non-labelled organs like pharynx or GFP-labelled entities could be segmented using HydraNets and assessed for their lifespan predictive ability using CAM approach and WormNet. It is tempting to speculate that akin to generative adversarial networks [36], future implementations of the by-design interpretability through a tandem of segmentation and classification may be trained end-to-end and employed for routine scientific discovery. The proof-of-principle automated analytical platform will be useful for non-invasive aging biomarkers discovery, particularly in young day 1–3 adult *C. elegans*. This has a great potential to accelerate the pharmaceutical screening for anti-aging drugs. The development of the methodology will also be helpful to find and characterize new pathologies in *C. elegans* important for basic aging research. To make the code available to the research community we have deposited it on GitHub (https://github.com/ails-institute/DeepLongevity).

## MATERIALS AND METHODS

### Code implementation

All the source code for this work was implemented in Python version 3.6, Tensorflow versions 1.9.0 or 2.3.0 [37] and Keras version 2.2.2 [38]. Tensors pre-processing and manipulation was implemented in Numpy 1.15.0 [39]. Python environment was maintained using anaconda distribution. Source code is available on GitHub (https://github.com/ails-institute/DeepLongevity).

### Model training

Training of WormNet, and fine-tuning of InceptionV3 were performed on a desktop PC equipped with Intel Core i7-8700K CPU at 3.7 GHz and 32 GB of RAM as well as GeForce 1080 Ti GPU. Training of U-Net and HydraNet were performed using Google Collaboratory cloud GPU (e.g., NVIDIA Tesla V100). Inference and analysis were performed using Google Collaboratory.

### Hyperparameters

WormNet used binary cross-entropy as a loss function and was trained using Adam optimizer with a starting learning rate of 0.005, beta 1 of 0.9, beta 2 of 0.999, epsilon of 1e-08, and decay of 0. A 30-fold data augmentation was performed for each epoch. U-Net and HydraNet were trained using Adam optimizer with a starting learning rate of 0.001 and default parameters. Dice loss function was used for the semantic segmentation task (see https://github.com/ails-institute/DeepLongevity).

### Statistical analysis

Statistical significance was evaluated using one-way ANOVA with Tukey’s HSD correction employing GraphPad Prism software.

### Architecture design and hyperparameters tuning

To ensure optimal performance of U-Net, WormNet, HydraNet and InceptionV3 architectures hyperparameters including, but not limited to the rate of learning, regularization through dropout coefficient were heuristically optimized. For the baseline comparison, all hyperparameters across similar architectures were kept comparable. In case of novel architectures, during the design process the expressive capacity or depth of the architecture was started at lowest applicable size and gradually increased until the point of performance convergence.

### The dataset

Raw images were deposited to BioImage Archive (EMBL): https://www.ebi.ac.uk/biostudies/studies/S-BIAD300.

## Supplementary Materials

Supplementary Figure 1
