# Supplementary material for: A tandem segmentation-classification approach for the localization of morphological predictors of C. elegans lifespan and motility
Source: Aging (Albany NY). 2022 Feb 25;14(4):1665–77. doi: 10.18632/aging.203916 (PMC8908923; doi:10.18632/aging.203916)
Supplement: Supplementary Figure 1 [file aging-14-203916-s001.pdf]

SUPPLEMENTARY FIGURE

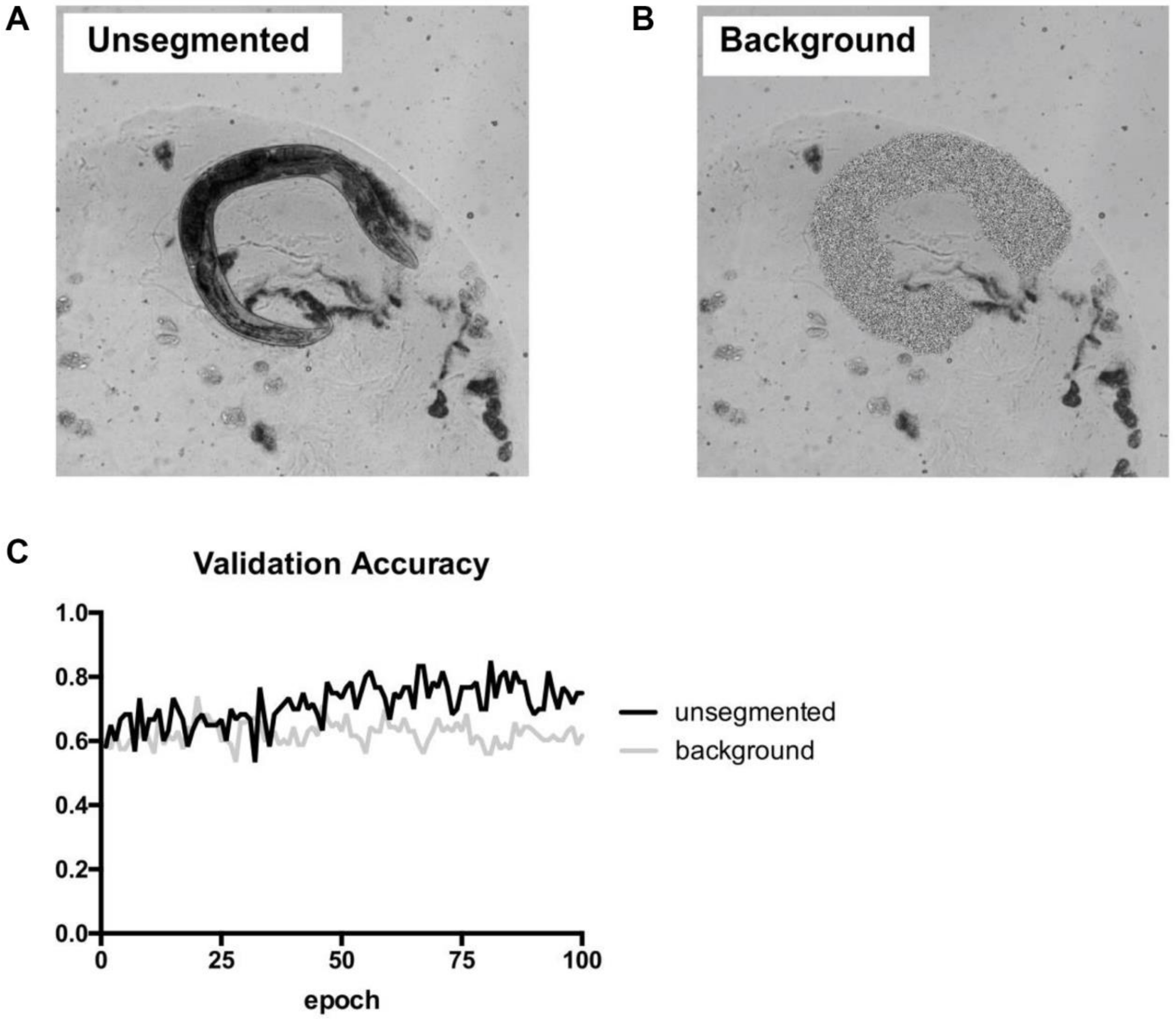

Supplementary Figure 1. Background contribution investigation. (**A**) Example of a raw (unsegmented) *C. elegans* micrograph. (**B**) Example of a micrograph with segmented *C. elegans* removed and substituted by a random noise – i.e., synthetic background. (**C**) Comparison of WormNet performance on an unseen validation dataset of unsegmented images (black) and synthetic background (grey).
